# Richmond Academy of Medicine and Surgery

**Published:** 1896-09

**Authors:** Landon B. Edwards

**Affiliations:** President, in the chair


### SOCIETY REPORTS.

### RICHMOND ACADEMY OF MEDICINE AND SURGERY.

*Regular Meeting, July* 14, 1896—*Dr. Landon B. Edwards, President, in the chair.*

Dr. Wm. S. Gordon read a paper on "The Treatment of Typhoid Fever, with a Report of Cases." [See page 454.]

DISCUSSION.

Dr. J. W. Long asked the temperature of the baths used, and if they were the only antipyretics employed.

Dr. Gordon.—The temperature was a little below normal, and gradually reduced. He endeavored to reduce temperature by nature's method, through the skin. He certainly would not use the Woodbridge method in atypical cases, especially where constipation is marked.

Dr. Jacob Michaux wished to give some personal experience. He desires to call attention to the great good obtained from the use of strychnine. Benefit is derived through two channels: the effect upon the heart, the effect upon the nervous system, both being "toned."

One of the most important duties falling upon the doctor in typhoid fever is the maintenance of the integrity of the stomach: Take care of the stomach, and the patient can better bear disease, especially if the siege be a long one.

Another observation is, we are often too nervous about the diarrhea. It is eliminative, and unless the number of actions is sufficient to weaken the patient—say over six or seven a day—it is better to let it alone.

The subject of antipyretics in typhoid is an important one. He is afraid of coal-tar preparations, as they are too depressing. The administration of water is of vast importance—allow it freely for drinking purposes, as it aids the emunctories. When used externally, no antipyretic can compare with it, it reducing fever decidedly and safely.

He prefers the use of whisky to brandy—being cheaper and obtainable in a purer state. It should, however, be used cautiously, but persistently.

DR. GEO. BEN. JOHNSTON said Dr. Gordon's paper suggested some points upon which he wished to speak. He has no belief in the existence of typho-malarial fever. The reports of so-called typho-malarial fever are cases of atypical typhoid. It has been his experience that we see very few typical cases in this region, the majority being the so-called typho-malariæ, in which quinine produces little or no effect except to aggravate the insomnia of the early stage, increase nervousness, and but seldom appreciably reduce temperature. If the last is effected, the advantage is dearly bought.

Now, he never administers quinine beyond the point of testing the fever, and if no response is early elicited, he abandons it.

Regarding the coal-tar preparations, he agrees with Drs. Gordon and Michaux. No harm—on the contrary, much good can be obtained from the use of small doses of phenacetine in the early stage, for cephalalgia and the general aching. He would not continue its employment later, when the circulation is depressed. For the relief of epistaxis, a 10 per cent. solution of antipyrine sprayed in the nostrils is efficacious.

Regarding turpentine, it is an old friend, and physicians are too apt to overlook it. Dr. Gordon is right in valuing it, being especially useful in the later stages.

Comparing first, the old depletive treatment, large doses of calomel and copious bleeding, and second, the expectant plan, with the modern method, the essence of the latter-day success may be summed up as—1, absolute rest in the recumbent posture, nothing is more dangerous than to allow the patient to sit up even for stool; 2, abundance of such food as will sustain and not irritate, viz.: sweet and buttermilk, thin broth, and copious draughts of pure water which carries off effete material; 3, thorough ventilation, even in winter.

DR. EDWARD MCGUIRE believes in little medicine; many cases can be treated without it. It is most important to preserve the stomach. He wishes to go on record as opposed to all antipyretics except water. For the first two days he gives quinine for diagnostic purposes.

Regarding rest, he agrees with Dr. Johnston, for we can never tell, even in mild cases, the extent of typhoid lesions. He impressed the importance of administering abundance of liquids, and referred to Pepper's success with nitrate of silver.

Dr. J. A. Hodges.—Cases vary so much that it is difficult to establish any routine plan of treatment. His method may be divided into—1. Nutritive and tentative, comprising nourishment, rest, attention to the *primæ viæ*, strychnine, and digitalis; for diarrhea, beta-naphthol, and copper arsenite with morphine. He has never seen the latter combination fail. 2. Modified Brandt which he administers himself. No coal-tar product should be given in a disease whose tendency is to devitalize. 3. Chlorine treatment, which he has used since it was first introduced. For meteorism, sordes, tympanites, and diarrhea, he has not seen the time when it has not done more than turpentine.

Dr. Hugh M. Taylor thought in transfusion of normal saline solution we have an agent of great potency in treating the depression of typhoid fever which threatens life by the heart. Within the past week, this had been impressed upon him. In the seventh week of fever, a young woman was apparently dying from exhaustion. Was cold and sweating and unconscious from great depression. Respiration, 30 or 40; pulse, 140, and absent at the wrist. The continuance of life seemed a matter of only an hour or two. Whisky and strychnia, and digitalis and caffein, and nitro-glycerin had been pushed as rapidly as was thought safe, with no perceptible influence for good. A quart or more of saline solution in the cellular tissue of the thigh and shoulder became diffused by massage, but apparently was not taken up by the vascular vessels; certainly produced no appreciable effect upon the heart. Such was the undistended condition of the blood-vessels, even after cording up the arm, that it was difficult to find a suitable vein. Into the open vein the sharp point of the glass pipette of an eye-dropper was fastened by a ligature, and the other end of the pipette was fastened in the end of the long tube attached to a fountain syringe. This quickly improvised transfusion apparatus acted nicely. By the time as much as a pint of fluid had been introduced into the vein the pulse was full and strong, and the introduction of a quart in all increased the volume and arterial tension until the moribund patient's pulse was stronger than that of any of the attendants. The skin became dry, the color to the face and lips more natural, and the extremities warm. Not much improvement in the breathing was noticed; and while the volume of the pulse was so much improved, its rapidity was not correspondingly decreased. The pulse-rate continued very rapid, and the respiration rapid and shallow.

The improvement, after the transfusion, lasted five or six hours, at which time the pulse lost in volume until the wrist-pulse was indistinguishable. Transfusion direct was again resorted to with the same encouraging result of increasing the arterial tension and drying the skin, but no effect upon the pulse-rate or the respiration was apparent. This last function became more and more embarrassed, and was the ultimate cause of death.

His experience in this case impresses him with the belief that we have in transfusion a means of great value in the depression incident to hemorrhage in typhoid fever, and possibly an adjunct in the depressed circulation resulting from any one or more of the many complications arising in typhoid fever.

DR. J. W. LONG agrees with the treatment given by Dr. Gordon with the exception of digitalis. The criterion of its value is the effect in the case under observation. In some, it acts well; in others, it makes the heart weaker.

Regarding shock sometimes observed in administering the bath, it may be obviated by constant friction to the extremities while the patient is in the water.

DR. ARTHUR JORDAN adds his evidence as to the success of the chlorine treatment. He believes that in the course of time the antiseptic method will be the accepted one. He thinks that the patient is often over-fed, verified by the tympanites and the appearance of indican in the urine, indicating fermentation in the bowel.

Diagnosis may be made by microscopic examination of the blood.

In the first week, there is an increase of polynuclear cells in cases where complications, as of lung and kidney disease, may ensue. In the second week, there is an increased lymphocytosis diagnosing typhoid from other infectious diseases. In every instance, the nucleus of the lymphocyte is larger than that of the leucocyte, indicating nature's method of cure. Another condition is the marked increase of polynuclear cells, which, if occurring in the third week, shows suppurative processes to be abnormally increased.

Dr. J. W. Henson said the action of digitalis is uncertain because of the uncertainty of the preparation.

Dr. George Ben Johnston said the Brandt method is the most satisfactory antipyretic measure. The danger line of typhoid is a temperature of 103°. He has seen a temperature of 104° reduced in fifteen minutes to normal; he has seen it kept down for twelve hours; has seen it rise again in two hours.

Dr. Landon B. Edwards thought the abuse of some of the coal-tar derivatives had been emphasized too much in this discussion. It was undoubtedly because the gentlemen had used too large doses of phenacetin, antipyrine, etc., seeking too immediate results. It is too much forgotten that while large or even beyond moderate doses of phenacetin—anything more than four or five grains—reduces arterial pressure, *small doses increase and maintain the same.* Doses of this drug not exceeding two or three grains, every three or four hours, so as not to exceed twenty grains a day, are safe, and usually effective in keeping the temperature down to 101° or 102°. In moderate doses it will not reduce the temperature beyond the normal. It is a curious fact that it acts best in reducing fever temperatures during the hours after ten or eleven o'clock in the day, and there is not much to be expected of its after-midnight administration. There is no objection to combining it with strychnine or digitalis, as he often does. He almost invariably combines it with some antiseptic, such as salol. Like salol, phenacetin is taken up chiefly from the small intestines. And it is not improbable that its long-continued use in small doses favorably influences the typhoid ulcers of the small intestines; for the value of phenacetin, as of antipyrine, as a local application to ulcers in general, is too well known by practitioners to need emphasizing. Between these doses of phenacetin, he has come to rely with much confidence upon the "chlorine treatment," as described many years ago by Watson, later by Murchison, and more recently by Yeo. In a broad-neck twelve-ounce bottle, put fifteen grains of chlorate of potash; add slowly one drachm of strong hydrochloric acid. The characteristic green vapor develops. Add gradually an ounce or two of distilled water, pouring it down the side of the bottle, and put in the stopper so as to let none of the chlorine vapor escape. After awhile whatever quantity is not absorbed by the water settles on its surface. Then gradually add more water, and so proceed until the bottle is filled with water. These details as to the preparation are important; for, as Mr. Hugh Blair remarks, a different chemical result follows if water is added in advance of the hydrochloric acid. Dose of this mixture, an ounce every two to four hours. If the taste is disagreeable, add an ounce of syrup of orange peel to the twelve-ounce chlorine mixture. Yeo claims that 24 to 36 grains of soluble quinia added to this mixture helps its antifebrifuge action; but Dr. Edwards has been better satisfied with the phenacetin in the manner spoken of. As to the chlorine mixture, it promptly cleans the tongue; it perfectly deodorizes the bowel discharges in two or three days, and, especially when combined with the phenacetin treatment, it reduces the febrile temperature; its maintains physical strength and mental clearness; assists in assimilation of food; acts its part as an antiseptic, and shortens the average course of the fever. Of course such is not the full line of treatment, for recumbency, quiet of surroundings, composure of mind, the sufficient use of water as a drink, and the topical use of cool water sponging or wet packs, the supply of proper nutritious fluid diet, etc., are essential. Half the battle, it may be, depends on the faithful, well-trained nurse; but fully the other half depends on the able, experienced, and ever-watchful, judicious doctor who does not become a routinist. For the past eight years Dr. Edwards has adopted the general line above described, with the loss of a single typhoid patient; and that one, in July, 1891, was a case in which he did not follow out the principles above spoken of. That case set in as a profuse hemorrhagic case. Incidentally he remarked that *early* profuse, repeated typhoid hemorrhagic cases are those that appear to set at defiance the results of any plan of treatment yet devised. In comparing his results with those who adopt the Brandt or the Woodbridge, or any other specific plan of treatment, he sees no reason to prefer a change of base. Carter, Czerna, and others long ago pointed out that *small doses* of phenacetin increase arterial tension; while all agree that *large doses* reduce the same, and that it acts directly on the thermogenic centers, lessening the production of animal heat and slightly decreasing heat dissipation. Of course these remarks are intended to discuss but a partial phase of the important subject of treatment of typhoid fever, and are brought out only because of the surprising disfavor in which coal-tar antipyretics seem to be held by some of the speakers to-night.

In closing the discussion, Dr. Gordon congratulated the Academy on the unanimity of opinion regarding the subject under discussion, and was gratified to have his own views corroborated by the expressions of the different speakers.

He hoped that Dr. Jordan and others, who were devoting special attention to the examination of blood in disease, would continue a work which, to a greater or less extent, will give us the priceless advantage of accurate diagnosis in the early stages of fever and other morbid conditions. He looked for great discoveries in this department of pathology.

Personally he had expressed himself as not unwilling to use the coal-tar antipyretics, when indicated in the first days of fever by a sufficiently strong pulse and high temperature. Several patients in the group had been given phenacetin with benefit. And so he agrees with Dr. Edwards that the drugs in question may prove of great service if administered in judicious doses and under justifying circumstances.

With regard to digitalis, Dr. Long had misunderstood the tenor of his remarks, which was to prove that this drug may serve a good purpose by slowing and consequently strengthening an exhausted and rapidly-beating heart. This agent stimulates the inhibitory vagus, and its action can, as a rule, be controlled equally as well as a great many other agents are controlled. It must be reliable, and, when used generally, be administered when the patient is recumbent, in order to obtain the best effects. He cited the case in which its effects were so marked as a commentary upon Dr. Osler's remark that, personally, he is not convinced that digitalis does good in the failing heart of fevers. The results observed from its use alone, or combined with strychnine, will sometimes be entirely satisfactory. He would not continue it unless its physiological action was produced within a reasonable length of time.

Dr. Taylor's case, in which he had been a willing and profited assistant, was well worthy of mention. He had never before seen so prompt a relief of the symptoms of collapse; and the possibilities of direct transfusion in conditions which were favorable to permanent benefit from this measure were difficult to estimate.

He would be glad to use the chlorine treatment so highly lauded by Drs. Hodges and Edwards, and intended to do so when opportunities presented for this or any other special antiseptic remedy. In referring to turpentine, the design was only to emphasize the numerous indications occurring at the same time which could be met with this familiar but none the less valuable drug.

Dr. Pepper's statement (quoted by Dr. Edward McGuire), that one hundred consecutive cases of typhoid fever occurring in private practice and first seen, as a rule, in the early stages, had been successfully treated with nitrate of silver alone or in combination; and that under this invariable treatment in all private cases, for a period of ten years, no death had occurred, was well worthy of consideration, coming from so high an authority. Dr. Frederick P. Henry, of Philadelphia, had commented, however, upon Dr. Pepper's statement, referring to the fact that the cases were in private practice, and that the unusually excellent results were doubtless largely owing to the great advantage of early and good hygienic and medicinal treatment which Dr. Pepper's patients enjoyed.

DR. GORDON said, in conclusion, that, in his opinion, relapses were due, in the vast majority of cases, to dietetic indiscretions or to a too early rising from bed. It was quite true that relapses did occasionally occur when no special cause could be assigned; but, in his experience, relapses had been owing to the disregard of his injunctions as to diet and exercise. Too much stress could not be laid upon this point.

